# βIV spectrin abundancy, cellular distribution and sensitivity to AKT/GSK3 regulation in schizophrenia

**DOI:** 10.1038/s41380-025-02917-1

**Published:** 2025-02-07

**Authors:** Jessica Di Re, Michela Marini, Syed Ibrar Hussain, Aditya K. Singh, Akshaya Venkatesh, Musaad A. Alshammari, Tahani K. Alshammari, Abdul-Rizaq Ali Hamoud, Ali Sajid Imami, Zahra Haghighijoo, Nickolas Fularcyzk, Laura Stertz, Derek Hawes, Angela Mosebarger, Jordan Jernigan, Claire Chaljub, Ralda Nehme, Consuelo Walss-Bass, Anton Schulmann, Marquis P. Vawter, Robert McCullumsmith, Robert D. Damoiseaux, Agenor Limon, Demetrio Labate, Michael F. Wells, Fernanda Laezza

**Affiliations:** 1 Department of Pharmacology & Toxicology, University of Texas Medical Branch at Galveston, Galveston, TX USA; 2 Department of Mathematics, University of Houston, Houston, TX USA; 3 MD-PhD Combined Program, University of Texas Medical Branch at Galveston, Galveston, TX USA; 4 Department of Pharmacology & Toxicology, College of Pharmacy, King Saud University, Riyadh, 11451 Saudi Arabia; 5 Department of Neurosciences and Psychiatry, University of Toledo College of Medicine and Life Sciences, Toledo, OH USA; 6 Louis A. Faillace Department of Psychiatry and Behavioral Sciences, McGovern Medical School, University of Texas Health Science Center, Houston, TX USA; 7 Stanley Center for Psychiatric Research, Broad Institute of MIT and Harvard, Cambridge, MA USA; 8 Department of Stem Cell and Regenerative Biology, Harvard University, Cambridge, MA USA; 9 Department of Psychiatry, Columbia University Irving Medical Center, New York State Psychiatric Institute, New York, NY USA; 10 Functional Genomics Laboratory, Department of Psychiatry & Human Behavior, University of California, Irvine, Irvine, CA USA; 11 Neurosciences Institute, Promedica, Toledo, OH USA; 12 Department of Molecular and Medical Pharmacology, University of California Los Angeles, Los Angeles, CA USA; 13 Department of Bioengineering, University of California, Los Angeles, CA USA; 14 California NanoSystems Institute, University of California, Los Angeles, CA USA; 15 Jonsson Comprehensive Cancer Center, University of California, Los Angeles, CA USA; 16 Department of Neurology, University of Texas Medical Branch at Galveston, Galveston, TX USA; 17 Mitchell Center for Neurodegenerative Diseases, University of Texas Medical Branch at Galveston, Galveston, TX USA; 18 Department of Human Genetics, David Geffen School of Medicine, University of California Los Angeles, Los Angeles, CA USA; 19 Molecular Biology Institute, University of California Los Angeles, Los Angeles, CA USA

**Keywords:** Schizophrenia, Neuroscience

## Abstract

Schizophrenia (SCZ) is a complex psychiatric disorder with unclear biological mechanisms. Spectrins, cytoskeletal proteins linked to neurodevelopmental disorders, are regulated by the AKT/GSK3 pathway, which is implicated in SCZ. However, the impact of SCZ-related dysregulation of this pathway on spectrin expression and distribution remains unexplored. Here, we show that βIV spectrin protein levels were reduced in neurons of the dorsolateral prefrontal cortex in SCZ postmortem samples compared to healthy control (HC) from the Human Brain Collection Core (HBCC). To investigate potential links between βIV spectrin and the AKT/GSK3 pathway, we analyzed the PsychEncode dataset, revealing elevated SPTBN4 and AKT2 mRNA levels with correlated gene transcription in both HCs and individuals with SCZ. Next, computational tools were employed to identify potential AKT and GSK3 phosphorylation sites on βIV spectrin, and two GSK3 sites were validated through in vitro assays. To assess whether βIV spectrin distribution and sensitivity to AKT/GSK3 are altered in SCZ, we used iPSC-derived neurons from two independent cohorts of patients with significantly increased familial genetic risk for the disorder. Alteration in βIV spectrin levels and sensitivity to AKT/GSK3 inhibitors were consistently observed across both cohorts. Importantly, a Random Forest classifier applied to βIV spectrin imaging achieved up to 98% accuracy in classifying cells by diagnosis in postmortem samples, and by diagnosis or diagnosis × perturbation in iPSC samples. These findings reveal altered βIV spectrin levels and AKT/GSK3 sensitivity in SCZ, identifying βIV spectrin image-based endophenotypes as robust, generalizable predictive biomarkers of SCZ, with the potential for scalable clinical applications.

## Introduction

Schizophrenia (SCZ) is a severe mental illness characterized by significant impairments in cognition, perception, and behavior, affecting nearly 1% of the population [1, 2]. Despite high heritability of SCZ, the mechanisms underlying the complex interplay between genetic and environmental factors that influence its clinical presentation remain poorly understood. While a limited number of studies provide evidence of specific genes involved in SCZ [3, 4], extensive genome-wide association studies and differential transcriptomic analyses in case-control studies have revealed numerous minor variations in gene expression and genetic variants, each with a small, cumulative effect on disease risk [5–9]. These variations have been found to converge on highly preserved gene hubs that are associated with synaptic function, cell-adhesion, intracellular signaling pathways, and neurotransmitter balance in neurodevelopment [8–12]. However, the mechanisms linking vulnerable gene hubs to molecular endophenotypes of the disease at the cellular level are not clear.

Spectrins, a diverse class of cytoskeletal proteins, play a pivotal role in regulating the cellular architecture of neurons through interactions with F-actin [13, 14]. In neurons, β spectrins are the master structural elements of the axon initial segment (AIS), a specialized subcellular compartment crucial for neuronal firing [15–18]. Specifically, βIV spectrin directly interacts with voltage-gated Na+ (Nav) channels, controlling their precise localization at the AIS and modulating neuronal firing in response to stimulation and/or intracellular kinase signaling, a phenomenon known as AIS plasticity [19–24]. Loss of βIV spectrin disrupts the cytoarchitecture of the AIS and leads to impaired temporal fidelity of action potentials [25] making it susceptible to disruptions associated with disease [26].

Previous research has demonstrated that the AKT/GSK3 signaling pathway regulates the distribution of βIV spectrin in primary hippocampal neurons [27, 28]. AKT directly phosphorylates and inhibits GSK3 through serine phosphorylation at S21/9 (GSK3αS21/GSK3βS9 [29]. Inhibition of AKT increases distribution of βIV spectrin at the AIS and dendrites, which is associated with increased intrinsic firing of hippocampal neurons [27]. Given the extensive literature on the involvement of the AKT/GSK3 pathway in SCZ [30–35], these findings provided the basis for investigating whether: (i) the distribution of βIV spectrin is altered in SCZ; (ii) βIV spectrin is a target of the AKT/GSK3 pathway; and (iii) the sensitivity of βIV spectrin to the AKT/GSK3 pathway is altered in SCZ.

Confocal imaging of βIV spectrin in postmortem tissue from patients with SCZ revealed a lower level of βIV spectrin compared to healthy controls (HC). We also conducted targeted differential analyses of βIV spectrin transcripts using a data set from the PsychENCODE Knowledge Portal, which—after controlling for confounding factors—revealed differentially expressed βIV spectrin in SCZ. Computational algorithms predicted four peptide sequences in βIV spectrin as targets of the AKT/GSK3 pathway, which we further analyzed using an atlas of human kinases, lending additional support that βIV spectrin is a target of the AKT/GSK3 pathway [36]. This was followed by an in vitro test of kinase activity, which showed that GSK3 phosphorylates peptides within βIV spectrin. Finally, two separate sets of neurons derived from induced pluripotent stem cells (iPSCs) from patients with SCZ were used to assess βIV spectrin protein levels and determine its sensitivity to AKT/GSK3 pathway inhibition. These experiments uncovered a loss of βIV spectrin regulation by AKT and GSK3 in iPSC neurons from patients with SCZ. An unbiased machine learning approach to analyze these imaging datasets identified generalizable predictive patterns in neurons from patients with SCZ from both *postmortem* and iPSC neuron samples. Combined with the well-known role of the AKT/GSK3 pathway in SCZ [31, 32], these studies underscore the involvement of βIV spectrin and its regulation via AKT/GSK3 phosphorylation in endophenotypes associated with SCZ.

## Materials and methods

### Derivation of human induced pluripotent stem cell-derived neurons

Two sets of experiments using human iPSC induced neuronal cell lines were performed. The first experiment was performed in iPSC induced neurons from a Central Valley of Costa Rica (CVCR) SCZ multiplex family, for which sample selection and derivation have been previously described [34, 37, 38]. The iPSC lines were originally derived from lymphoblastoid cell lines. Subjects were thoroughly characterized in prior studies, following the Principles of the Declaration of Helsinki. To maintain anonymity, all cell-lines were de-identified. For these studies, neuronal cell lines from five siblings in the multiplex family, two healthy control (HC) and three SCZ, were chosen to achieve the most homogenous genetic background possible for functional studies.

The second was performed using two healthy control (HC) cell lines from the California Institute of Regenerative Medicine (CIRM) biobank managed by FujiFilm Cellular Dynamics. Two SCZ cell lines were generated from patients diagnosed with SCZ and harboring microduplication of the 16p11.2 chromosomal region. From these patients, iPSCs were generated through collaboration between the Karolinska Institute (Solna, Sweden) and the Broad Institute (Cambridge, MA). Further information is included in Supplemental Table 1.

### Cell culture

Culturing of iPSC induced neurons from a Central Valley of Costa Rica (CVCR) SCZ multiplex family, including reprogramming of human lymphoblastoid cell lines (LCLs) into human induced pluripotent stem cells (hiPSCs), neuronal precursor cell (NPC) differentiation, and cortical neuron differentiation, has been previously described [34]. Briefly, LCLs were reprogrammed into hiPSCs using the Epi5™ Episomal iPSC reprogramming Kit. Cells (2 × 10^6) were nucleofected with episomal vectors and cultured in RPMI1640 medium for 3 days before transferring to Matrigel-coated dishes. After colony formation (15–30 days), clones were expanded and one clone per subject was differentiated into hiPSC-derived neuronal precursor cells (NPCs) using AggreWell™ methodology. Embryoid bodies (EBs) were formed and cultured in STEMdiff™ Neural Induction Medium, followed by rosette selection and expansion in Neurobasal medium with FGF until passage 3. hiPSC-derived NPCs were differentiated into cortical neurons by cultivation on Laminin/Poly-L-Ornithine-coated plates in Neurobasal Medium supplemented with BDNF, GDNF, dibutyryl-cyclic AMP, ascorbic acid, IGF-1, and WNT-3A.

16p11.2 microdeletion SCZ patient and corresponding HC cells lines were plated on Geltrex basement membrane matrix (1:100; Life Technologies, A1413301) and maintained in mTesR Plus (Stemcell Technologies, #100-0276) medium. Cells were split every 4–5 days via a 10 minute/37 °C incubation in Accutase (Innovative Cell Technologies, AT104) followed by 1:5 dilution in mTeSR Plus. Plating medium was supplemented with ROCK inhibitor Y-27632 (10 µM; Stemgent, 04-0012) for 12–24 h. Stem cells were transduced with doxycycline-inducible Ngn2 and expanded as previously described [39]. Transduced iPSCs were plated on Geltrex (1:100) for neural induction as previously described [40]. In brief, stem cells were fed with Induction Medium: DMEM/F12 (ThermoFisher, 11320082), Glutamax (1:100; ThermoFisher, 10565018), 20% Glucose (1.5% v/v), N2 Supplement (1:100, ThermoFisher, 17502048), LDN-193189 (200 nM; Stemgent, 04-0074), SB431542 (10 mM; Tocris, 1614), XAV939 (2 mM; Stemgent, 04-00046), and Doxycycline (2 mg/mL; Sigma-Aldrich, D9891). After 24 h, cells were fed with Induction & Selection Media (Days 2-5): DMEM/F12, Glutamax (1:100), 20% Glucose (1.5% v/v), N2 Supplement (1:100), puromycin (5 mg/mL; ThermoFisher, A1113803), LDN-193189 (100 nM), SB431542 (5 mM), XAV939 (1 mM), and Doxycycline (2 mg/mL). On day 6, cells were fed with Induction & Selection Media with the addition of FUDR (10uM; Sigma Aldrich F0503). The next day, cells were passaged in Accutase and plated on PDL/Laminin-coated 96-well plates at 40,000 cells/cm2 in Neuron Medium: Neurobasal Medium (ThermoFisher 21103049), 20% Glucose (1.5% v/v), Glutamax (1:100), MEM-NEAA (1:100; Life Technologies, 10370088), B27 supplement (1:50; ThermoFisher 17504044), BDNF (10 ng/mL; R&D Systems 248-BDB), GDNF (10 ng/mL; R&D Systems 212-GD), and CTNF (10 ng/mL; R&D Systems 257-NT). Neurons were fed every 3–4 days until Day 21 post-induction.

### Inhibitor treatment

On Day In Vitro (DIV) 21, neurons were treated with GSK3 Inhibitor CHIR99021 (20 μM; Tocris, Minneapolis, MN), AKT inhibitor triciribine (50 μM; SelleckChem, Houston, TX), or 0.5% DMSO control. After 24 h of treatment, cells were fixed and processed for imaging.

### Immunocytochemistry

A detailed method of staining for proteins at the AIS or in neurites in vitro has previously been published [34, 41]. Briefly, the following primary antibodies were used to stain proteins of interest: mouse monoclonal IgG2b against βIV spectrin (1:200, NeuroMabs, Davis, CA. Cat. 75–377) and chicken polyclonal against MAP2 (1:10000, Invitrogen, Carlsbad, CA. Cat. pa1-10005). In some instances, an additional AIS marker such as neurofascin (Mouse monoclonal IgG1, 1:100, Neuromab Cat. 75-027) was included. Isotype specific secondary antibodies against mouse and chicken primary antibodies (Alexa 488, Alexa 647, Invitrogen) were used at a 1:200 concentration.

### Immunohistochemistry

Slide mounted *postmortem* dorsolateral prefrontal cortex (DLPFC) (Brodmann’s area 46) samples were obtained from the National Institutes of Mental Health Human Brain Collection Core (HBCC). Samples came from SCZ subjects and HC (no history of psychiatric disorder) and were matched according to *postmortem* interval (PMI), tissue pH, age at death, and RNA integrity number (RIN). Samples from Caucasian and African American males were included. No difference was seen in age between HC (38.47 ± 14.65 years) and SCZ (45.83 ± 14.59 years) or in PMI (HC: 31.67 ± 18.16 h; SCZ: 40.13 ± 18.83 h) as analyzed by Mann-Whitney test. Samples with level of education higher than bachelor’s degree and suicide as cause of death were excluded. A full description of sample demographics is provided in Supplemental Table 2.

Staining procedures were previously described [42]. In brief, following 20 min tissue fixation in 1% formaldehyde (a dilution of 37% formaldehyde solution in PBS, MasterTech Scientific, catalog number fxfor37gal), tissue was permeabilized using 1% Triton X-100, 0.5% Tween-20 in PBS for 7–10 min. Primary antibodies used in the *postmortem* experiment were mouse monoclonal IgG2b against βIV spectrin (1:500 NeuroMab, catalog number 75-377) and guinea pig polyclonal against NeuN (1:250, Synaptic System, catalog number 266 004).

### Image acquisition

CVCR neuronal cultures and *postmortem* tissue images were acquired with a Zeiss LSM-880 with Airy scan confocal microscope with a 63X oil immersion objective (1.4 NA). Multi-track acquisition was done with excitation lines at 488 nm for Alexa 488, 561 nm for Alexa 568 and 633 nm for Alexa 647. Z-stacks were obtained every 0.43 μm with a frame size of 1024 ×1024 pixels and a scan speed of 0.77 μs. The same pinhole setting was used for all channels.

16p11.2 microdeletion SCZ patient and corresponding HC cell images were acquired on a Molecular Devices ImageXpress Confocal in widefield mode equipped with a custom Nikon 60x Plan Fluor Objective (0.85 numeric aperture) and a Zyla sCMOS camera with 4.2 megapixel, no binning. Four sites per well with 25 images with a step size of 400 nm. We imaged optical stacks containing DAPI, FITC, TRITC and CY5 channels for a total of 100 images per site, 400 images per well. Each site was individually autofocused using IR laser focusing. All images were automatically saved into an SQL database with MetaXpress frontend, exported as 16 bit TIFF files with a metadata text file for further processing.

### Image analysis

All images were analyzed using the open-source ImageJ (http://imagej.nih.gov/ij) or Fiji software (https://fiji.sc/) for processing fluorescence intensity. Analysis was performed by blinded experimenters. For soma fluorescence intensity analysis, Z-stacks of confocal images were sum-projected, a region of interest (ROI) corresponding to the soma was highlighted using an intensity threshold method, and mean fluorescence intensity within the ROI was quantified [27, 41]. Quantification of the fluorescence intensities of AIS (CVCR, *postmortem* tissue) and neurite (16p11.2 microdeletion) staining was performed using a method previously described in [43]. In brief, the fluorescence intensity of the AIS was analyzed by drawing a segmented line of 5 pixels wide along the AIS, starting from the soma on the overlay image with any AIS crossing over somas or other cell neurites being excluded. Similarly, the fluorescence intensity of neurites was analyzed by choosing one neurite per cell approximately 5 pixels wide that did not cross over any other neurites or somas and tracing the longest path for each selected neurite.

### Machine learning analysis

AIS intensity measures were first calibrated to ensure that all signals were computed starting at the same anatomical location, corresponding to the beginning of the AIS, and then truncated, if needed, to ensure that all traces had the same pixel length. Neurite intensity measures were similarly calibrated by truncation, if needed, to ensure that all traces had the same pixel length. To select the features for the AIS and neurite intensities, we computed the Discrete Fourier Transform (DFT) of the fluorescence intensity signal along each AIS and neurite. A Random Forest classifier was trained using the DFT coefficients of the fluorescence intensity signal to predict either treatment (for the cultured neurons experiment: CHIR, TRI, DMSO) or the disease status (for the *postmortem* experiment: SCZ vs HC). To select intensity features for the soma analysis, a mask was generated for each soma region, then statistical matrices were computed from the pixel values in the masked soma region using the publicly available py-Radiomics toolbox [44]. Based on the seminal work by Haralick on the Gray-Level Co-occurrence Matrix [45], we created a statistical matrix with 14 py-radiomics features including the Gray-Level Dependence Matrix and the Gray-Level Size Zone. A Random Forest classifier was trained using these py-radiomics features to predict either treatment (for the cultured neurons experiment: CHIR, TRI, DMSO) or the disease status (for the *postmortem* experiment: SCZ vs HC).

For each classification model above, the classifier was trained using 70% of the samples with the remaining 30% of the samples used for testing. For each classification, the experiment was repeated 10 times. Training and testing samples were randomly swapped in each iteration to ensure the stability of the classifier and prevent a single choice of training and testing samples from introducing bias. A more detailed description of our analysis, including examples of the DTF coefficients computed from the AIS and/or neurite signals, are provided in the Supplementary Methods.

### Transcriptome data analysis

For RNA-seq data from *postmortem* human dorsolateral prefrontal cortex (DLPFC), gene-level counts were downloaded from the PsychENCODE Knowledge Portal on synapse.org. Data from the CommonMind Consortium (CMC), HBCC, Lieber Institute for Brain Development (LIBD) and BrainGVEX study were included [6]. Analysis was limited to neurotypical controls and individuals with SCZ, age of death of 17 years or older (age of youngest SCZ case), and where RIN and PMI were available. In case of technical replicates, the sample with the higher RIN value was used. Analysis was limited to genes expressed above 5 counts per million (CPM) in at least 10 samples. This threshold was chosen to ensure that the genes considered in the differential gene expression test were expressed at a high level in at least a small number of samples. Differential gene expression was analyzed using limma/voom [46] and surrogate variable analysis (SVA) [47] with the following model: *Gene expression ~ psychiatric diagnosis + brain bank + biological sex + age of death* + *3 surrogate variables*. The number of surrogate variables was chosen based on splitting the datasets into three subsets (CMC, HBCC/LIBD, and BrainGVEX) and correlating effect sizes for SCZ across the transcriptome, while varying the number of surrogate variables between 1 and 20. Sources of variations, including batch effects/ brain bank of origin, demographic variables, and surrogate variables were accounted for in the model. The highest Pearson’s and Spearman’s correlations were found with 3 surrogate variables. For heatmaps and pairwise gene correlations, residualized logCPM values were obtained in limma by regressing out all the covariates above except psychiatric diagnosis.

### Peptide prediction

Predicted phosphorylation sites were calculated using previous methods [48] and additionally analyzed using the human Phosphosite Plus kinase library [36]. A more detailed description of phosphosite analysis is provided in the Supplemental Methods.

### GSK3β kinase activity assay

The phosphorylation activity of β-IV spectrin peptides (PEP S-45: PAASTAAASLFECSRIK, PEP S-2543: RWGQTLPTTSSTDEGNPKR) and GSK3 peptide (H-YRRAAVPPSPSLSRHSSPHQ-pSer-EDEEE-NH2, positive control) against GSK3 kinase inhibitor CHIR99021 was evaluated using a GSK3β kinase activity assay kit (BPS Bioscience, San Diego, CA) according to the manufacturer’s protocol. Experiments were carried out in 96-well plates with 6 wells for each treatment. The enzymatic activity of GSK3β was measured using the Kinase-Glo reagent (Promega, Madison, WI) as a detection reagent. Luminescence was detected using the Synergy™ H4 Multi-Mode Microplate Reader (BioTek, Winooski, VT, USA).

### Statistical analysis

Normality was first assessed using QQ plots for each dataset and log transformed if necessary. Average fluorescence intensity of neurites and soma for each condition were analyzed using a Two-Way mixed model ANOVA with subject as the random effect to account for within-subject variations. This was followed by a Dunnett’s (within groups) or Sidak’s (between groups) multiple comparisons test for neuronal cultures to determine differences in βIV spectrin in neurites and soma. A nested t-test was used to determine differences between HC and SCZ in *postmortem* brain tissue, which also accounts for within-subject variation in the statistical model. These analyses were performed using GraphPad Prism 9. Transcriptome analysis was conducted in R, and p-values were adjusted for multiple testing using the Benjamini-Hochberg method.

## Results

### βIV spectrin is downregulated in the DLPFC of patients with SCZ

**Fig. 1:**
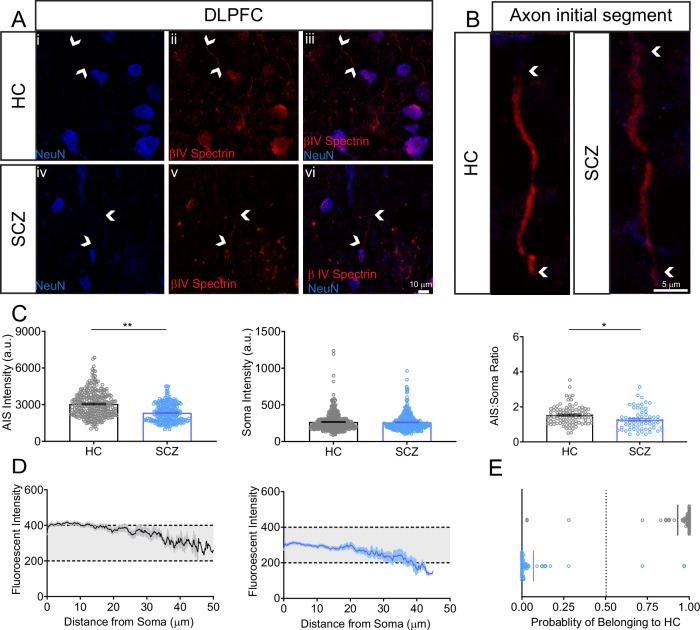
βIV spectrin AIS immunofluorescence is decreased in SCZ *postmortem* DLPFC. **A** Staining of NeuN and βIV spectrin in DLPFC L3b of HC and patients with SCZ. Scale bar in **A-vi** is 10 μm. **B** Zoom to AIS from **A** Scale bar is 5 μm. **C** Changes in βIV spectrin fluorescence intensity signal at the AIS (n = 288 HC, 243 SCZ), soma (n = 92 HC, 66 SCZ) and ratio of both measurements (n = 85 HC, 66 SCZ). **D** Profile of fluorescence intensity of βIV spectrin at the AIS of HC (gray) and SCZ (blue). **E** Accuracy of random forest classifier sorting of AIS fluorescence intensity. *p < 0.05 and **p < 0.01 by nested t-test (two-tailed) from n = 15 HC, 12 SCZ subjects. Each dot represents an individual cell measurement.

To evaluate whether changes in βIV spectrin are detectable in brains of SCZ patients, we first investigated whether there are alterations in βIV spectrin levels and distribution in *postmortem* brain tissue samples collected from individuals diagnosed with SCZ and HC. Fresh frozen tissue samples including the DLPFC were obtained from the NIMH HBCC. From the available samples, we selected n = 15 HC and n = 12 SCZ cases based on patient demographics and the overall suitability of tissue conditions for IHC. Our results showed a unique distribution pattern of βIV spectrin in neurons from HC samples, with perinuclear accumulations in the cytoplasm and enrichment in the AIS (Fig. 1A, B), closely resembling the distribution of the corresponding cultured neuronal protein [27]. An ROI-based analysis revealed a significant decrease in βIV spectrin fluorescence intensity at the AIS of SCZ patients compared to HC (Fig. 1C, p = 0.0072 by nested t-test), without changes in the fluorescence intensity of the protein in the soma (Fig. 1C). This also led to a significant reduction in the AIS/soma ratio (Fig. 1C, p = 0.0197 by nested t-test), which is typically used as a marker of neuronal polarity. It was noted that neurons from SCZ samples exhibited a less organized pattern in the soma with a pronounced accumulation in the AIS (Fig. 1Aii and Av). To determine if these changes allowed for accurate disease prediction, we applied a Random Forest classifier as in a previous study [38]. Here, we used βIV spectrin intensity features from the AIS and soma to better identify differences across multiple spatial scales [49]. Our findings showed that the extracted single cell features at both the soma and the AIS accurately predicted the disease class (Fig. 1E). Classification accuracy was 98.6% ± 1.1% the AIS, while changes in the soma were less reliably identified, albeit at an accuracy close to 63%. Overall, these results indicate that changes in fluorescence intensity signals related to βIV spectrin are reliably associated with SCZ diagnosis.

### βIV spectrin mRNA is upregulated in SCZ

**Fig. 2:**
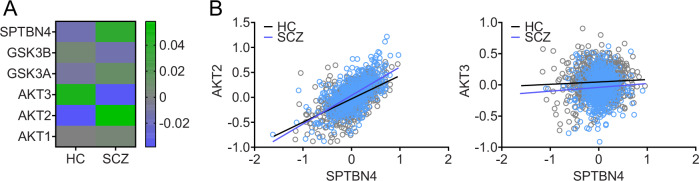
SPTBN4 and AKT2 upregulation in SCZ DLPFC with correlated expression. **A** Heatmap showing covariate-corrected mRNA expression of SPTBN4 and genes known to be involved in its modulation in the PsychEncode dataset (n = 802 HC, 563 SCZ). **B** Linear correlation of SPTBN4 with AKT2 was significant in HC (r2 = 0.309; p < 0.0001) and SCZ (r2 = 0.39; p < 0.0001). Each dot represents a single case.

To evaluate whether changes of βIV spectrin are observed in SCZ and support our immunofluorescence analysis in *postmortem* tissue, we investigated whether these disruptions are also evident at the mRNA level in a large cohort using the PsychEncode dataset, which includes mRNA sequencing data from 802 HC and 563 SCZ cases [6]. To control for potential confounding effects, we corrected for brain bank, sex, age and three surrogate variables as described in Methods. This analysis found that SPTBN4 is significantly elevated in SCZ (adjusted p = 0.003) compared to HC (Fig. 2A), suggesting that the decrease in βIV spectrin protein seen in *postmortem* tissue from the DLPFC (Fig. 1A–C) inversely correlates with changes in mRNA levels. As we hypothesized that *SPTBN4* expression levels may be modulated by AKT/GSK3 signaling, we also evaluated the expression of *AKT1*, *AKT2*, *AKT3*, *GSK3A* and *GSK3B*. We found that *AKT2* was increased in SCZ (p adjusted = 4.6 e-10) while *AKT3* was reduced (p adjusted = 8.8 e-12). Interestingly, *SPTBN4* expression was correlated with *AKT2* (Fig. 2B) suggesting that altered AKT/GSK3 signaling in SCZ may affect βIV spectrin. We did not detect significant changes in GSK3A or GSK3B, which supports the idea that the regulation of GSK3 activity in SCZ may result from transcriptomic changes in its upstream regulators, such as AKT, rather than direct transcriptomic changes in GSK3 itself.

### Prediction of AKT/GSK3 phosphosites on βIV spectrin

**Fig. 3:**
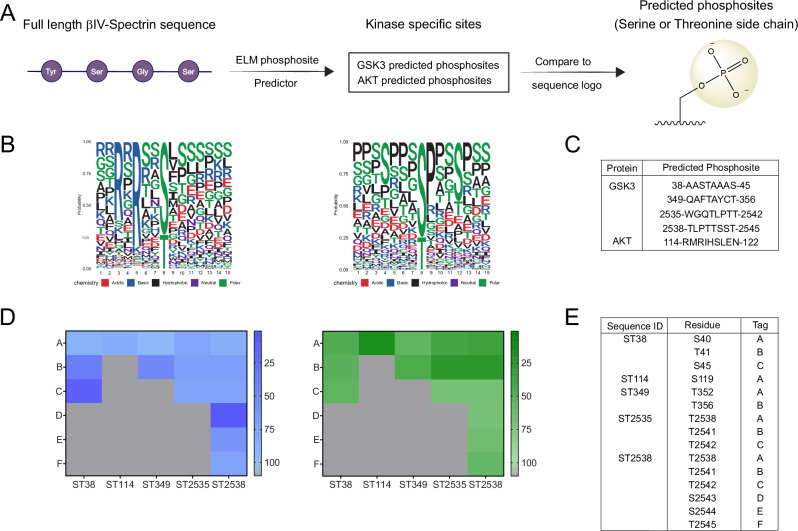
Multiple predicted GSK3 and AKT phosphosites in βIV spectrin. **A** Scheme of phosphosite prediction pipeline using βIV spectrin sequence and eukaryotic linear motif phosphosite predictor. **B** Predicted peptide sequence logos for GSK3 (left) and AKT (right). Amino acids are colored based on their chemical composition. Red - acidic, blue - basic, black - hydrophobic, purple – neutral, and green - polar. **C** βIV spectrin sequences predicted to be phosphorylated by either GSK3 or AKT. **D** Additional in silico prediction of phosphosites using the Phosphosite Plus Kinase Library showing likelihood of phosphorylation by GSK (blue) or AKT (green). Darker color indicates greater likelihood of phosphorylation of specific residues by GKS3 or AKT (ranked 1–100, 1 = highest likelihood). Gray squares indicate no phosphorylation sites at specific residues **E**. Matching key for ranked sites in **D**.

Ample evidence exists to support a key role of disrupted kinase pathways in SCZ [50]. In line with our results, AKT was previously found upregulated in the DLPFC of patients with SCZ [32], suggesting potential mechanistic links between disease endophenotypes and altered AKT pathway. However, as of yet, there are no recognized AKT/GSK3 phosphorylation sites on βIV spectrin. Thus, we used computational platforms to predict AKT/GSK3 phosphorylation sites on βIV spectrin (Fig. 3A). Using these methods, we used consensus sequences of βIV spectrin to predict potential phosphosites for both AKT and GSK3 (Fig. 3B) finding four potential phosphosites for GSK3 and one potential phosphosite for AKT (Fig. 3C). To validate these potential phosphosites as targets of either GSK3 or AKT, sequence-based kinase predictions were made for each of these sequences within βIV spectrin and ranked based on their likelihood (percentile rank) to be phosphorylated by either GSK3 or AKT using the human Phosphosite Plus kinase library [36] (Fig. 3D).

**Fig. 4:**
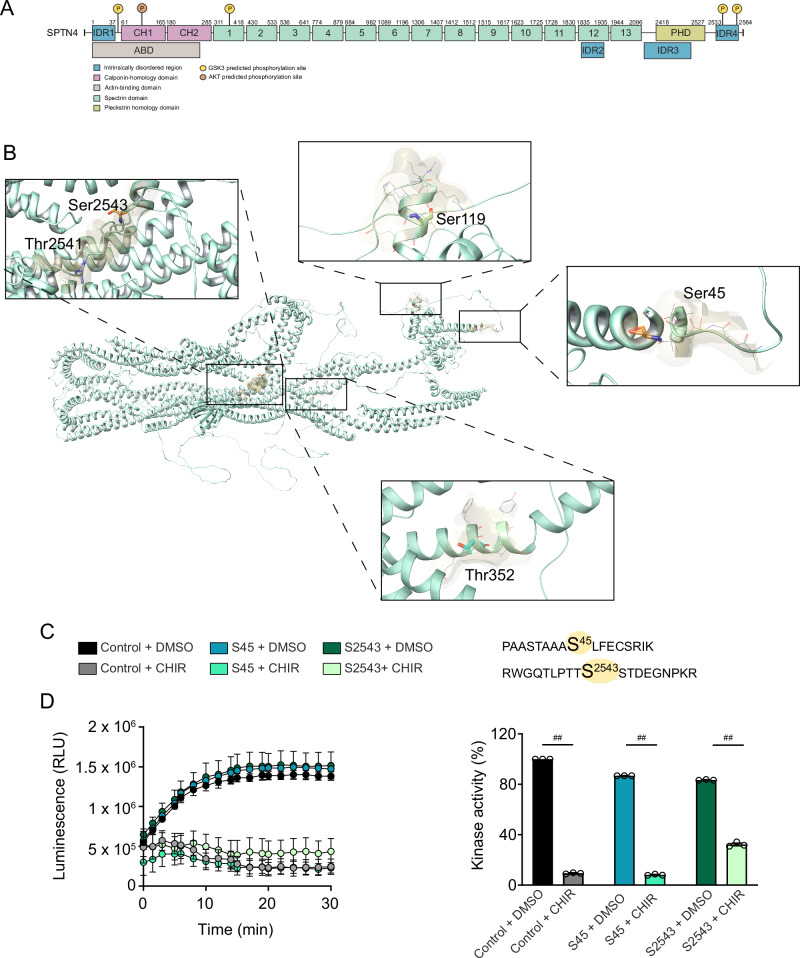
AlphaFold prediction and in vitro phosphorylation of GSK3 sites in βIV spectrin. **A** Full sequence of βIV spectrin and predicted phosphosite locations. Predicted GSK3 phosphosites (S45, T352, S2543 and T2541) are shown in yellow. The predicted AKT phosphosite (S119) is shown in orange. **B** Predicted phosphosite residues within AlphaFold structure of βIV spectrin are depicted at S119 (top), S45 (right), T352 (bottom), S2543 and T2541 (left). **C** Sequence of peptides used for in vitro phosphorylation. **D** Real-time luminescence from the Kinase-Glo reaction showing GSK3β activity in presence of control peptide (gray), βIV spectrin peptide containing S45 (teal) and βIV spectrin containing S2543 (green) in the presence of vehicle (darker colors) and CHIR99021 (lighter colors), with corresponding summary bar graphs (n = 3 reactions per tested condition). **p < 0.01 t-test. Each dot represents an independent reaction, each with 6 technical replicates.

Based on this analysis, we next mapped the predicted phosphosites onto the domains βIV spectrin. Three of GSK3’s potential phosphosites were within intrinsically disordered regions and one within the spectrin domain (Fig. 4A). AKT had one potential phosphosite within the calponin homology domain (Fig. 4A). Using AlphaFold structure modeling, each of the phosphosites was visualized on βIV spectrin (Fig. 4B). The potential AKT phosphorylation site, S119, was excluded for further testing, due to its position within an inaccessible portion of the protein. Among the four predicted GSK3 phosphorylation sites, S45 and S2543 were selected for experimental validation based on their high ranking in the Phosphosite Plus kinase library (Fig. 3D) and predicted accessibility from AlphaFold. To validate whether GSK3 phosphorylates either S45 or S2543 on βIV spectrin, we conducted an in vitro phosphorylation assay in the presence of GSK3β, the most relevant GSK3 isoform in SCZ [51], using two βIV spectrin peptides (Fig. 4C), containing either S45 (15-mer peptide) or S2543 (19-mer peptide), both alone and in the presence of the GSK3 inhibitor CHIR99021 (CHIR). Purified GSK3β efficiently phosphorylated a GSK3 control substrate peptide (positive control) included in the GSK3 kinase activity assay kit, as well as the two βIV spectrin peptides containing S45 and S2543 (Fig. 4D). These reactions were significantly inhibited by CHIR (Fig. 4E, t-test p < 0.01). Overall, these findings suggest that the AKT/GSK pathway likely regulates βIV spectrin through GSK3 phosphorylation of either the S45 or S2543 residue.

### Altered AKT/GSK3 regulation of βIV spectrin protein levels in SCZ neurons

One advantage of using neurons differentiated from iPSCs is the ability to test their response to pharmacological treatments, though they do not represent mature neurons or the complexity of human brain tissue. Thus, we aimed to determine whether the previously reported regulation of protein distribution at the AIS and in developing neurites by the AKT/GSK3 pathway [27, 28, 34], along with the disruptions to this pathway in SCZ [34], converge mechanistically and can be replicated in neurons derived from SCZ patients. To address this question, we differentiated neurons from two different groups consisting of HC and patients with SCZ. In the first group, both HC and SCZ cell lines were derived from a multiplex family of the Central Valley of Costa Rica (CVCR), previously used for interrogating mechanisms associated with SCZ [37, 52–60]. The second group consisted of two SCZ cell lines from patients with a 16p11.2 microduplication and two HC.

**Fig. 5:**
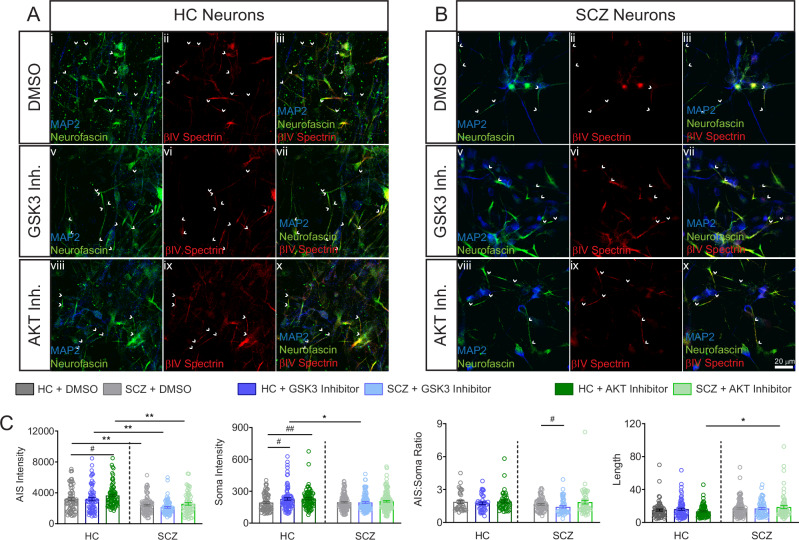
βIV spectrin abundance and sensitivity to AKT/GSK3 inhibition is altered in SCZ iPSC neurons from the CVCR multiplex family. **A** Staining of MAP2, βIV spectrin, and AIS marker (neurofascin) in neurons derived from HC iPSCs treated with vehicle (DMSO), GSK3 inhibitor (20 μM CHIR99021), or AKT inhibitor (50 μM triciribine). **B** Staining of MAP2, βIV spectrin, and AIS marker in neurons derived from SCZ iPSCs. Scale bar in (**Bx**) indicates 20 μm. **C** Effect of kinase inhibition on βIV spectrin intensity at the AIS (n = 66 HC DMSO, 93 SCZ DMSO, 72 HC GSK3 inh., 68 SCZ GSK3 inh., 76 HC AKT inh., 64 SCZ AKT inh.), soma (n = 70 HC DMSO, 136 SCZ DMSO, 91 HC GSK3 inh., 109 SCZ GSK3 inh., 72 HC AKT inh., 128 SCZ AKT inh.), AIS:soma ratio (n = 36 HC DMSO, 65 SCZ DMSO, 39 HC GSK3 inh., 52 SCZ GSK3 inh., 44 HC AKT inh., 49 SCZ AKT inh.), and length of staining at the AIS. # p < 0.05 and ## p < 0.01 by two-way mixed model ANOVA with Dunnett’s multiple comparisons test (within group effect of inhibitor vs. DMSO). *p < 0.05 and **p < 0.01 following two-way mixed model ANOVA with Sidak’s multiple comparisons test (between-groups comparison of inhibitor treatment). All data are from n = 2 HC cell lines and n = 3 SCZ cell lines. Each dot represents an individual cell measurement.

βIV spectrin expression pattern was characterized under basal conditions and in response to pharmacological inhibitors of the AKT/GSK3 pathway to assess its sensitivity to perturbations of this pathway. iPSC-neurons from CVCR subjects were cultured and fixed for immunofluorescence staining against βIV spectrin and MAP2 (Fig. 5A, B). The CVCR neurons showed an accumulation of βIV spectrin, indicative of an AIS, allowing for examination of changes in βIV spectrin accumulation across and between different subcellular compartments. As previously described in rat primary hippocampal neuron cultures [27], the intensity of βIV spectrin staining at the AIS is increased in HC neurons treated with the AKT inhibitor triciribine compared the HC neurons treated with vehicle DMSO, a response not seen in the SCZ cell lines (Fig. 5C, p = 0.0186). Between groups analysis also showed that the fluorescence intensity of βIV spectrin at the AIS was decreased in all SCZ cells treated with DMSO, the GSK3 inhibitor CHIR and the AKT inhibitor triciribine (Fig. 5C, p = 0.0048, p < 0.0001, and p < 0.0001 respectively by Two-way mixed model ANOVA). Additional measurements of βIV spectrin response to kinase inhibition were conducted by measuring the fluorescence intensity of βIV spectrin in the soma (Fig. 5C) and the ratio of βIV spectrin in the AIS and soma (Fig. 5C).

Treatment with CHIR or the AKT inhibitor triciribine increased the fluorescence intensity of βIV spectrin in the somas of HC (Fig. 5C, p = 0.0217, and p = 0.0044 respectively by Two-way mixed model ANOVA). However, the effect of both inhibitors was blunted in neurons from patients with SCZ, reaching statistical significance in case of CHIR (Fig. 5C, HC vs SCZ, p = 0.0493 by Two-way mixed model ANOVA). Differences were also observed in the AIS/soma ratio in SCZ neurons treated with CHIR, which showed a decrease compared to the SCZ DMSO group (Fig. 5C, p = 0.0322 by Two-Way ANOVA). Additionally, when examining the length of βIV spectrin staining as a measure of overall AIS length, differences were observed between HC and SCZ neurons in response to the AKT inhibitor (Fig. 5C, p = 0.0114 by Two-Way ANOVA). The AIS/soma ratio and AIS length are interrelated parameters: the ratio measures axonal polarity, while AIS length reflects axonal plasticity. These parameters indicate the neuron’s ability to traffic proteins to and move them along the axon, relying on coordinated cytoskeletal regulation [61]. Since AKT inhibition leads to GSK3 disinhibition, the aberrant responses to GSK3 and AKT inhibition in SCZ suggest a broader dysregulation of the neuronal cytoskeleton associated with the disease, which is in line with recent studies [62].

**Fig. 6:**
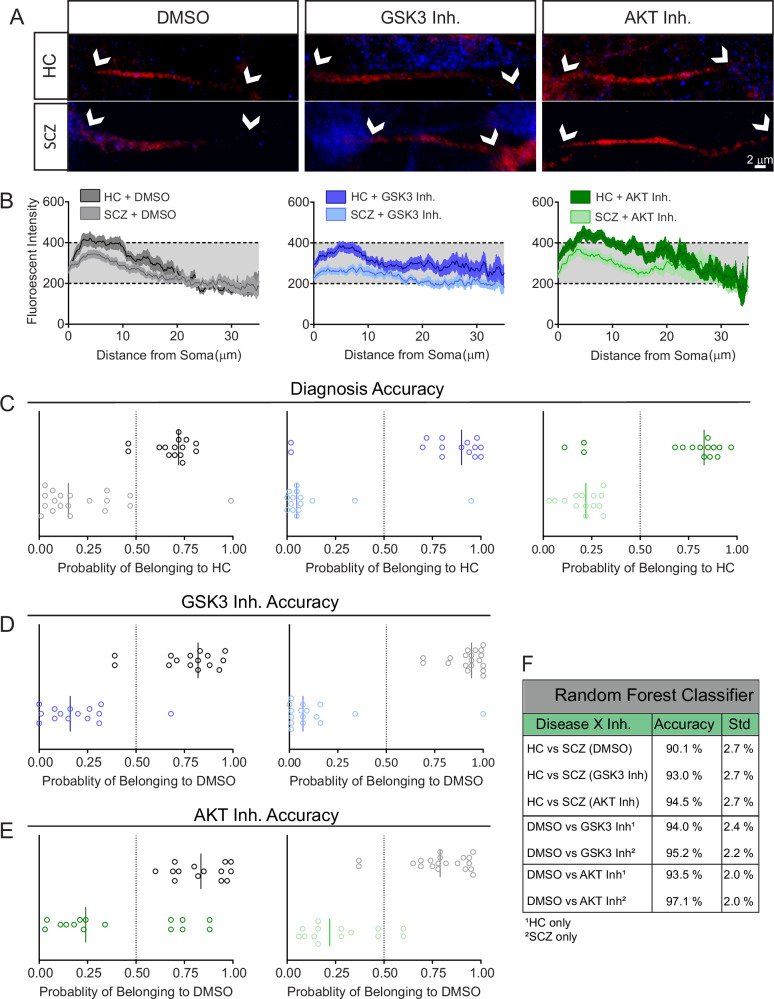
Accurate SCZ classification with Random Forest classifier of βIV spectrin image features. **A** Zoom to AIS of HC and SCZ neurons treated with vehicle, GSK3 or AKT inhibitor showing MAP2 (blue) and accumulation of βIV spectrin (red) at the AIS. White arrows indicate beginning and end of AIS ROI and the scale bar is 2 μm. **B** AIS fluorescence intensity signal of βIV spectrin. **C** Classification accuracy based on sorting signals between HC and SCZ groups for DMSO, GSK3 inhibitor, and AKT inhibitor-treated neurons. **D** Classification accuracy based on sorting signals between DMSO and GSK3 inhibitor-treated cells for HC and SCZ neurons, respectively. **E** Classification accuracy based on sorting signals between DMSO and AKT inhibitor-treated cells for HC and SCZ neurons, respectively. **F** Table of classification accuracy of all groups. The highest sorting accuracy was from SCZ neurons treated with AKT inhibitor.

These findings were supported by Random Forest classifier of the AIS fluorescence signal (Fig. 6A, B) which accurately separated iPSC-derived neurons based on βIV spectrin and kinase inhibitor perturbations. Classification accuracy for distinguishing HC vs SCZ neurons was similar at baseline (DMSO) and across both inhibitor treatments (Fig. 6C). Likewise, sorting accuracy for both HC and SCZ groups was greater than 93% in both the GSK3 inhibitor treated neurons (Fig. 6D) and the AKT inhibitor treated neurons (Fig. 6E). All sorting accuracies are summarized in Fig. 6F.

**Fig. 7:**
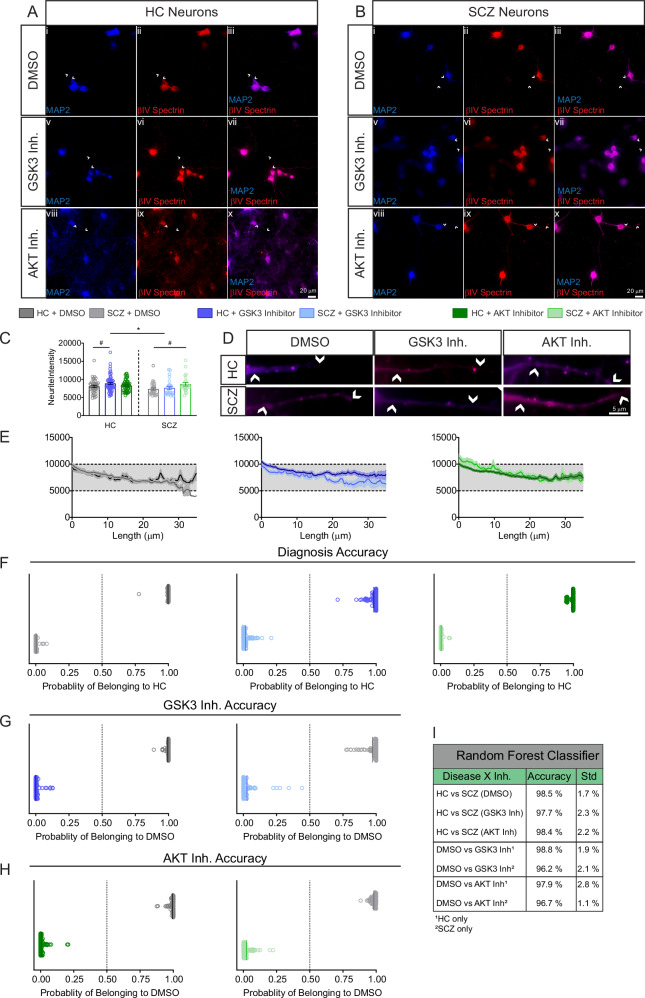
βIV spectrin abundance and sensitivity to AKT/GSK3 inhibition is altered in SCZ iPSC neurons from 16.2 microduplication patients. **A** Staining of MAP2 and βIV spectrin in neurons derived from HC iPSCs treated with vehicle (DMSO), GSK3 inhibitor (20 μM CHIR99021) or AKT inhibitor (50 μM triciribine). Scale bar in Ax is 20 μm. **B** Staining of MAP2 and βIV spectrin in neurons derived from SCZ patients with a 16p11.2 microduplication. Scale bar in Bx is 20 μm. **C** Effect of kinase inhibition on βIV spectrin fluorescence in neurites (n = 43 HC DMSO, 31 SCZ DMSO, 61 HC GSK3 inh., 23 SCZ GSK3 inh., 56 HC AKT inh., 24 SCZ AKT inh.). **D** Zoom to neurites of HC and SCZ neurons treated with vehicle, GSK3 or AKT inhibitor stained with MAP2 (blue) and accumulation of βIV spectrin (red) at the AIS. White arrows indicate beginning and end of neurite ROI and the scale bar is 5 μm. **E** Fluorescence intensity signal of βIV spectrin in neurites. **F** Classification accuracy based on sorting signals between HC and SCZ groups for DMSO, GSK3 inhibitor and AKT inhibitor-treated neurons. **G** Classification accuracy based on sorting signals between DMSO and GSK3 inhibitor-treated cells for HC and SCZ neurons, respectively. **H** Classification accuracy based on sorting signals between DMSO and AKT inhibitor-treated cells for HC and SCZ neurons, respectively. **I** Table of classification accuracy of all groups. # p < 0.05 by two-way mixed model ANOVA with Dunnett’s multiple comparisons test (within group effect of inhibitor vs. DMSO). *p < 0.05 and following two-way mixed model ANOVA with Sidak’s multiple comparisons test (between groups comparison of inhibitor treatment). All data are from n = 2 HC cell lines and n = 2 SCZ cell lines from patients with 16p11.2 microduplication. Each dot represents an individual cell measurement.

To assess reproducibility and consistency of the βIV spectrin phenotypes in other genetic models of SCZ, we repeated the experiments in iPSC-derived neurons from patients carrying a chromosome 16p11.2 microduplication which is associated with a 14.5 fold increased risk of SCZ [63]. This increased risk may be driven by changes in kinase signaling [64] that ultimately converge, once again, on GSK3 signaling [65]. The 16p11.2 microduplication cells were also cultured and fixed for immunofluorescence staining against βIV spectrin and MAP2 (Fig. 7A, B). Unlike the CVCR neurons, these cells did not contain identifiable AIS, likely due to a more immature developmental stage compared to the CVCR neurons. Therefore, βIV spectrin immunofluorescence in response to AKT/GSK3 inhibition was examined in neurites. This analysis showed an increase in βIV spectrin fluorescence in response to GSK3 inhibition in HC compared to DMSO (Fig. 7C; p = 0.0314), while the SCZ patients showed an increase in response to AKT inhibition compared to DMSO in the same group (Fig. 7C; p = 0.0227). Overall, this led to a significant difference between HC and SCZ neurons in response to GSK3 inhibition (Fig. 7C; p = 0.0155). Insets and intensity profiles are illustrated in Fig. 7D, E. Notably, the change in βIV spectrin intensity in response to AKT inhibition in neurites (Fig. 7C) mirrors the AIS length dysregulation observed in CVCR neurons under the same conditions (Fig. 5C). This suggests that SCZ neurons lack the ability to regulate βIV spectrin distribution within cytoskeleton-rich subcellular compartments in response to GSK3 disinhibition. The Random Forest classifier analysis was also performed on these data. Classifier accuracy was even higher with sorting accuracy exceeding 95% in all tested conditions including differences in βIV-spectrin in HC vs SCZ reaching a 98.5 ± 2.8% in distinguishing the two groups (Fig. 7F–I). Overall, these results provide evidence for a potential convergence between βIV spectrin and the AKT/GSK3 pathway in SCZ and corroborate the value of using βIV spectrin imaging features as a disease predictor.

## Discussion

The AKT/GSK3 pathway has been associated with SCZ for nearly two decades, though the exact mechanisms by which it contributes to the disease remain elusive [32]. In contrast, the potential role of spectrins in SCZ is much newer, though emerging evidence suggests that pathogenic variants or differential expression of spectrins may contribute to the pathogenesis of not only SCZ, but also other neurodevelopmental disorders or spectrinopathies [7, 26, 66–71]. Transcriptional analyses of *postmortem* brain tissue have demonstrated that a subset of transcripts belonging to the spectrin protein family is severely disrupted in individuals with SCZ and may be linked to disease progression [72]. Spectrins are upregulated in the temporal cortex [11] and in the subgenual anterior cingulate cortex of patients with SCZ, even after correcting for cross-diagnostic and diagnosis-specific changes in RNA expression [7]. In a cross-sectional study of SCZ correlating gene expression to functional neuroimaging, βIV spectrin expression had a strong negative correlation with indicators of brain function in SCZ across disease progression [73]. The findings presented here not only add to the growing evidence that βIV spectrin is disrupted in the pathophysiology of SCZ, but also offer valuable insights into the molecular mechanisms that regulate its cellular distribution, which may be a factor underlying this complex mental disorder.

Here, we present evidence that βIV spectrin is disrupted in *postmortem* tissue at both the protein (Fig. 1) and transcript level (Fig. 2). Although the direction of effects differs between mRNA and protein studies—with mRNA levels increased and protein levels decreased—the fact that both are altered in SCZ is significant. In fact, discrepancies between mRNA and protein expression levels are common [74], highlighting the importance of evaluating both transcriptional and protein changes, particularly in disease states.

Previous studies reported a decrease in ankyrin G immunoreactivity, a key organizer of the AIS, in the DLPFC of SCZ patients, though no change was observed in the density (number per mm^2^) of βIV spectrin-immunoreactive AIS [75]. This discrepancy is likely due to differences in quantification methods (fluorescence intensity vs. AIS density) or confounding factors such as education level, which were controlled for in the present study but not reported in Cruz et al. [75]. Notably, Cruz et al. found that βIV spectrin density was unaffected by antipsychotic use or substance use history, which were not controlled for in the present study [75].

Previous studies have indicated a role of the GSK3/AKT pathway in regulating βIV spectrin at the AIS, but direct phosphorylation has not been confirmed [27]. Thus, we used a combination of in silico methods to identify potential a total of five potential phosphosites for GSK3 and AKT within βIV spectrin (Fig. 3). Narrowing down to two potential phosphosites, we showed phosphorylation of βIV spectrin by GSK3 (Fig. 4), which further supported testing functional correlations between βIV spectrin and the AKT/GSK3 pathway, which we examined in two separate cohorts of familial SCZ cases.

We used two cohorts of iPSC-derived neurons from patients with increased familial risk for SCZ: CVCR patients and those with 16p11.2 microduplication. In both cases, we found evidence of βIV spectrin altered pattern expression and sensitivity to AKT/GSK3 pathway dysregulation, indicating that βIV spectrin and AKT/GSK3 targets are convergent.

In the CVCR patients, we observed a blunted response of βIV spectrin at the AIS of neurons from patients with SCZ treated with GSK3 and AKT inhibitors when compared to their HC counterparts (Figs. 5, 6). This could suggest altered AIS plasticity due to dysregulation of the AKT/GSK3 pathway in SCZ. Evidence from in vitro studies and animal models, ranging from chicken embryos to rodents to nonhuman primates, indicates that the structure and composition of the AIS changes in response to stimulation and intracellular signaling [22, 25, 27, 28, 76–78]. In turn, this AIS plasticity affects neuronal firing properties, allowing neurons to fine-tune their output in response to stimulation [19, 20]. Our results indicate that inhibition of AKT in HC neurons increases the fluorescence intensity of βIV spectrin, a key component of the AIS, in a similar manner to what is seen in murine neurons [27]. In contrast, not only is βIV spectrin intensity lower in all tested conditions in the SCZ neurons compared to HC, but SCZ neurons lack a response to GSK3 and AKT inhibition.

In 16p11.2 microduplication patients, the baseline expression pattern of βIV spectrin in neurites did not reach statistical significance compared to HCs. However, HC neurons and SCZ neurons showed different responses to inhibition of GSK3 and AKT. As with the AIS and soma measurements from the CVCR neurons, the differences between HC and 16p11.2 microduplication SCZ neurons appeared in the GSK3 inhibited cells. In both cohorts of cells, this may be due to changes in kinases other than AKT which regulate the activity of GSK3 [34, 64, 65] suggesting that the blunted response of βIV spectrin to GSK3 inhibition may be a key phenotype in SCZ. Likewise, the Random Forest classifier calculated a 98.5% accuracy in distinguishing the two groups, indicating clear signal differences. This confirms that βIV spectrin is a reliable classifier for SCZ.

Taken together, the results from our neuronal culture experiments indicate that both sets of SCZ neurons have a blunted response of βIV spectrin to AKT/GSK3 kinase inhibitors, which may reflect an overall impairment in their ability to adjust their firing in response to intracellular signaling. In line with the present findings, SCZ neurons exhibit aberrantly active Nav channel function [79]. This could be the result of altered binding of βIV spectrin to specific Nav isoforms or altered developmental Nav isoform switch.

In neurons, βIV spectrin may be regulated by AKT/GSK3 through direct phosphorylation by GSK3 at S45 or S2543, as confirmed by the Phosphosite Plus library and kinase activity assays. While more studies are needed to confirm direct phosphorylation in neurons and its role in SCZ, our analysis strongly suggests that βIV spectrin is a target of GSK3 phosphorylation. AKT suppresses GSK3, and when AKT is inhibited by triciribine, GSK3 is disinhibited due to reduced S9/S21 (inhibitory) phosphorylation. This suggests that the effects of AKT or GSK3 inhibition may lead to opposite levels of GSK3 activity, influencing βIV spectrin through varying phosphorylation at S45 or S2543. Changes in βIV spectrin phosphorylation can alter its interaction with the cytoskeleton, affecting trafficking along the axon and neurites—regulations that are disrupted in SCZ [80]. However, other downstream targets may also be affected by AKT inhibition, as triciribine and CHIR99021 did not produce opposite effects on βIV spectrin in this or previous studies [27]. In HC neurons from the CVCR cohort, βIV spectrin intensity at the AIS is particularly sensitive to AKT inhibition, while in the neurites from the 16.2 microduplication, it appears more responsive to GSK3 inhibition. The consistent differences observed between HC and SCZ samples treated with a GSK3 inhibitor suggest that active GSK3 regulates βIV spectrin distribution, an effect that is blunted in SCZ cells. Overall, the results from our neuronal culture experiments are in line with decreased activity of GSK3 and increased AKT activity in SCZ [81–85], emphasizing the importance of looking at the activity of kinases within disrupted pathways, as GSK3 itself was not dysregulated at the transcriptomic level (Fig. 2). These results corroborate a critical role of the AKT/GSK3 signaling pathway in SCZ as a phenotype that is consistent across multiple patient populations and sample types.

We then applied Random Forest algorithms to evaluate the discriminative power of βIV spectrin imaging features in postmortem brain tissue and iPSC neurons. This analysis achieved over 95% accuracy, revealing robust βIV spectrin-based imaging phenotypes linked to diagnosis and diagnosis × perturbation conditions through precise predictions from image-based classifiers. Thus, SVM-based features defined by βIV spectrin imaging are generalizable SCZ classifiers. When applied to functional data from patient-derived iPSCs, integration of SVM-based classifiers may accelerate target identification for drug discovery and clinical diagnosis for complex and heterogenous disorders such as SCZ.

## Supplementary information

Supplemental Methods

Supplemental Table 1

Supplemental Table 2

## Data Availability

All data will be made available upon request.
