# Supplementary material for: βIV spectrin abundancy, cellular distribution and sensitivity to AKT/GSK3 regulation in schizophrenia
Source: Mol Psychiatry. 2025 Feb 7;30(7):3090–102. doi: 10.1038/s41380-025-02917-1 (PMC12185341; doi:10.1038/s41380-025-02917-1)
Supplement: Supplementary file 1 — Supplemental Methods [file 41380_2025_2917_MOESM1_ESM.docx]

**SUPPLEMENTAL METHODS**

MACHINE LEARNING ANALYSIS

Three experiments were conducted:

- Experiment 1 on cultured CVCR neurons. Goal: to predict treatment: CHIR vs TRICI vs DMSO
- Experiment 2 on postmortem tissue. Goal: to predict disease status, SCZ vs HC
- Experiment 3 on cultured 16p11.2 microduplication neurons. Goal: to predict treatment: CHIR vs TRICI vs DMSO

For each experiment, fluorescent intensity signals were collected at the AIS and the soma. We described below how we carried out the analysis of the AIS data and of the soma data.

- AIS data analysis

To analyze the fluorescent intensity signals measured at the AIS, we proceeded as follows.

1. We first calibrated the intensity measures at the AIS to ensure that all signals were computed starting at the same anatomical location, corresponding to the beginning of the AIS and then truncated the measurements, whenever was needed, to ensure that all traces had the same length in pixel. Below are the details of the modifications

Experiment 1

|  | Original number of traces | Usable traces |
| --- | --- | --- |
| CHIR | HC: 75 SCZ:68 | HC: 47 SCZ:51 |
| TRICI | HC: 80 SCZ:65 | HC: 50 SCZ:46 |
| DMSO | HC: 71 SCZ:95 | HC: 51 SCZ:66 |

Experiment 2

|  | Original number of traces | Usable traces |
| --- | --- | --- |
| HC | 288 | 199 |
| SCZ | 243 | 172 |

Experiment 3

|  | Original number of traces | Usable traces |
| --- | --- | --- |
| CHIR | HC: 122 SCZ:136 | HC: 100 SCZ:100 |
| TRICI | HC: 115 SCZ:151 | HC: 107 SCZ:107 |
| DMSO | HC: 157 SCZ:178 | HC: 155 SCZ:155 |

1. We applied the Fourier transform (FFT) to each fluorescent intensity signal.
2. We computed fluorescent intensity features for each trace working on the signal in the Fourier domain. We found that this approach was significantly more effective (in terms of classification performance) than the alternative one where features are computed directly in the spatial domain. We attribute this behavior to the reduced sensitivity to quantization noise and the improved interpretability of the signal in the Fourier domain.

We selected 6 features from the python library tsfresh (they were found to be the most significant): abs_energy, mean_abs_change, absolute_sum_of_changes, calc_median, calc_max, rms

*abs_energy*: Returns the absolute energy of the time series which is the sum over the squared values

*mean_abs_change*: Average over first differences. Returns the mean over the absolute differences between subsequent time series values

*absolute_sum_of_changes:* Returns the sum over the absolute value of consecutive changes in the series

*calc_median:* Computes median of the signal

*calc_max:* Computes the maximum value of the signal

*rms:* Square root of the arithmetic mean (average) of the squares of the original values

We trained a Random Forest classifier using our single-cell fluorescent intensity features, to predict either (for the cultured neurons experiment) the treatment (CHIR, TRI, DMSO) or (for the postmortem experiment) the disease status (Scz vs HC). To compute our classification models we trained the classifier using 70% sample traces and using 30% traces for testing. Here are the detailed numbers.

**Experiment 1:**

**HCvsSCZ, CHIR fixed:** 68 sample traces and using 30 traces for testing

**HCvsSCZ, TRICI fixed:** 67 sample traces and using 29 traces for testing

**DMSOvsCHIR, HC fixed:** 68 sample traces and using 30 traces for testing

**DMSOvsCHIR, SCZ fixed:** 82 sample traces and using 35 traces for testing

**DMSOvsTRICI, HC fixed:** 71 sample traces and using 29 traces for testing

**DMSOvsTRICI, SCZ fixed:** 78 sample traces and using 34 traces for testing

**Experiment 2:**

**SCZvsHC**: 259 sample traces and using 112 traces for testing

**Experiment 3:**

**HCvsSCZ, CHIR fixed:**150 sample traces and using 64 traces for testing

**HCvsSCZ, TRICI fixed:**217 sample traces and using 93 traces for testing

**HCvsSCZ, DMSO fixed:**140 sample traces and using 60 traces for testing

**DMSOvsCHIR, HC fixed:**145 sample traces and using 62 traces for testing

**DMSOvsCHIR, SCZ fixed:**145 sample traces and using 62 traces for testing

**DMSOvsTRICI, HC fixed:**177 sample traces and using 78 traces for testing

**DMSOvsTRICI, SCZ fixed:** 178 sample traces and using 77 traces for testing

We repeated the classification analysis 10 times, randomly swapping the traces assigned to training and testing each time. This procedure was applied to ensure the stability of classifier and avoid that a single choice of training and testing samples could introduce a bias.

The images below show that differences of the fluorescent intensity traces at the AIS are more recognizable in the Fourier domain (i.e., after applying the FFT to the trace) shown in the top row with respect to the original representation in the bottom row.


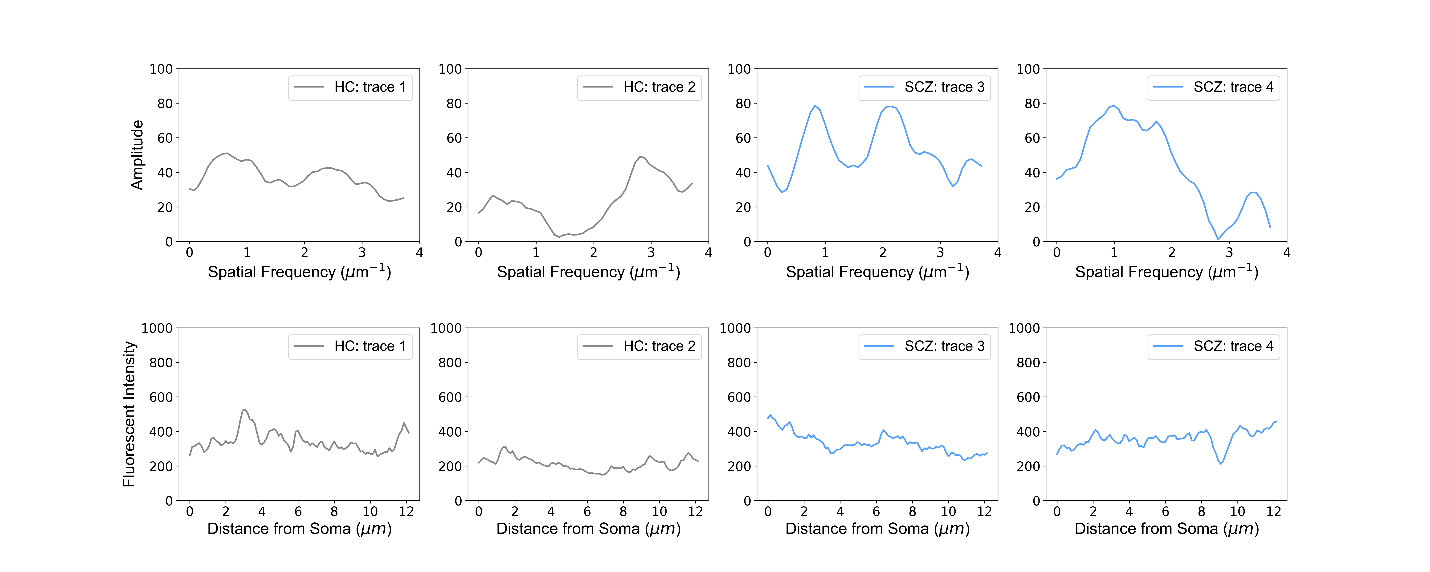


- Soma data analysis

To analyze the fluorescent intensity signals measured in the soma region, we proceeded as follows.

1. We first generated a mask for each soma region.
2. To compute image-based features to train our classifier we have chosen a class of statistical matrices derived from the pixel values in the soma region. Statistical matrices derived from gray-level pixel values have been used successfully in several image applications starting with the seminal work by Haralick on the Gray-Level Co-occurrence (GLC) Matrix [Har] and include the Gray-Level Dependence (GLD) Matrix, introduced for problems of texture classification, and the Gray-Level Size Zone (GLSZ) Matrix, proposed for applications in cell classification. All these matrix-based features are part of the current Image Bio-marker Standardization Initiative which aims to improve reproducibility and validation of radiomic features for applications in medical imaging. The numerical implementation of such matrices is publicly available in the py-Radiomics toolbox [Pyr]. Hence, we mapped each soma image into a vector containing 14 py-radiomics features.
3. We trained a Random Forest classifier using our py-radiomics features to predict either (for the cultured neurons experiment) the treatment (CHIR, TRI, DMSO) or (for the postmortem experiment) the disease status (Scz vs HC). [Total number of ROI SCZ: 110, ROI HC: 78] To compute our classification models, we trained the classifier using

70% sample traces and used 30% traces for testing or

*Experiment 2:*

132 sample traces and used 56 traces for testing

We repeated the classification analysis 10 times, randomly swapping the traces assigned to training and testing each time. This procedure was applied to ensure the stability of classifier and avoid that a single choice of training and testing samples could introduce a bias.

**Soma classification Results (PostMortem Only):**

|  | **Accuracy** | **Std** |
| --- | --- | --- |
| **HC vs SCZ**  (balancing data) | 62.66% | 0.00224 |

PEPTIDE PREDICTION

Seed data were generating by extracting the 5 phosphosties predicted to be phosphorylated by AKT1 or GSK3B using phospho.ELM and PhosphoSitePlus. The following table shows each sequence, along with the sequence ID used in the above figure and other relevant information.

| **Sequence ID** | **Sequence** | **Start Position** | **End Position** | **Implicated Kinase** |
| --- | --- | --- | --- | --- |
| ST38 | AASTAAAS | 38 | 45 | GSK3 |
| ST114 | RMRIHSLE | 114 | 121 | AKT |
| ST349 | QAFTAYCT | 349 | 356 | GSK3 |
| ST2535 | WGQTLPTT | 2535 | 2542 | GSK3 |
| ST2538 | TLPTTSST | 2538 | 2545 | GSK3 |

Seed data were then transformed by first identifying the canonical sequence (Q9H254) from UniProtKB. We then used a sliding window of +/- 5 Amino Acids to generate seven sequences, by moving the original sequence from right to left. The generated seven sequences were then processed to identify all potential residues that could be phosphorylated by a Serine/Threonine Kinase and then processed into the format suitable for input to the Kinase Library. The following table shows an example of the sequences generated by the sliding window for ST38.

| **Sequence ID** | **Sequence** |
| --- | --- |
| 32-WEREQPAASTAAASL-46-L15-ST38 | WEREQPAASTAAASL______ |
| 33-EREQPAASTAAASLF-47-L15-ST38 | _EREQPAASTAAASLF_____ |
| 34-REQPAASTAAASLFE-48-L15-ST38 | __REQPAASTAAASLFE____ |
| 35-EQPAASTAAASLFEC-49-L15-ST38 | ___EQPAASTAAASLFEC___ |
| 36-QPAASTAAASLFECS-50-L15-ST38 | ____QPAASTAAASLFECS__ |
| 37-PAASTAAASLFECSR-51-L15-ST38 | _____PAASTAAASLFECSR_ |
| 38-AASTAAASLFECSRI-52-L15-ST38 | ______AASTAAASLFECSRI |

A list of upstream kinases was generated by entering the transformed data into the kinase library with the following options:

- Do not account for phosphopriming
- Do not account for serine versus threonine favorability.

The resulting list for each sequence included 303 STKs in a ranked list, ordered by their percentile rank. For each set of seven lists for each sequence, we chose the median highest rank for each kinase to generate a consensus list of kinase ranking.
